# Commentary: Mutual interaction of basophils and T cells in chronic inflammatory diseases

**DOI:** 10.3389/fimmu.2016.00135

**Published:** 2016-04-08

**Authors:** Salvatore Chirumbolo

**Affiliations:** ^1^Unit of Geriatry, Department of Medicine, University Laboratory of Medical Research-LURM est, Policlinico GB Rossi, Verona, Italy

**Keywords:** leukotriene B4, cysteinyl leukotriene, basophils, allergy and immunology, T cells

Sarfati et al. recently published a thorough overview on the role exerted by basophils in the immune system and their relationship with T cells in chronic inflammation (1). In their paper, the authors fundamentally referred to the relationship with T cells, and basophil participation in innate and acquired immunity, through the complex network of immune cells and cytokines. Many further, though less appealing, mediators should be introduced, yet.

The paper by Sarfati et al. contains interesting bullet points on the biology of basophils, which deserve further insights and raise also some concern about the use and meaning of these cells in immunology laboratory (2). Allergy tests usually consider basophils as independent leukocytes able to elicit or regulate a typical type I hypersensitivity reaction, most commonly reported as an allergic response. Therefore, in this perspective, basophils are investigated fundamentally by the membrane recycling and up- or downregulation of IgE-induced surface displaced molecules, which undergo a turn over mechanism due to the basophil-mediated immune response. Actually, these cells play a pivotal role in the immune system and even their use in allergy diagnosis should be reappraised (3, 4). This would mean that basophils need to be treated not only as isolated cells responding to allergens but also as innate T-cells, quite neglecting their close interaction with other immune cells such as T-cells, platelets or other leukocytes. Yet, basophils appear to interact with a wide group of immune cells, either from innate or acquired immunity, particularly in chronic inflammation. In this scenario, lipid-derived molecules might play a major role also for basophils.

During allergy, leukocytes produce many mediators, some of which of recent interest, such as 15-hydroxyeicosatetraenoic acid (15-HETE) (5). This endogenous eicosanoid can interact with basophils, which possess receptors for 15-HETE (6) or bind to intracellular non G-coupled receptors such as PPAR-γ, which is expressed also in basophils (7). Aside from the overview described by the authors, this issue may be important during chronic allergic inflammation. Recent reports have shown that the activity of 15-lipooxygenase type 1 (15-LO-1) is fundamental in causing pathophysiology of asthma. During an inflammatory or physical injury, human airway epithelial cells increase their 15-LO-1 activity and the production of 15-HETE, besides the production of eoxin C4 (EXC4) or 14,15-leukotriene C4, from arachidonic acid (8). Basophils express cysteinyl leukotriene receptors (CysLTR) (9), CysLTR1 and 2 are upregulated in macrophages by IL-4 and IL-13 (10) and interact each other to regulate mitogenic signaling responses in mast cells (11). Cysteinyl leukotrienes (CysLTs) are important molecules produced by basophils, eosinophils, mast cells, and macrophages during innate immunity and in this sense represent important molecules to be focused when talking about basophils involvement in chronic inflammation (12). The authors questioned of how basophils are recruited to the site of inflammation and referred to CRTH2 (PGD2 receptor) and IL-3 receptor as possible target molecules for recruitment (1). At least for eosinophils, evidence suggested that CysLRRs are associated with the recruitment of these leukocytes in the site of allergic inflammation (13). To date, no information was reported about the possible involvement of these receptors in basophil diapedesis but this issue deserves major interest, due the role exerted in eosinophils. Basophils express LTC_4_, which is consequently transformed into LTD_4_ and the more stable LTE_4_ and in this sense they actively participate in asthma and control, through CysLTRs the induction of a Th2-mediated response to allergens (14). Actually, past data from cysteinyl leucotriene receptors antagonists confirm the role of these mediators in allergic asthma (15). This perspective may encourage researchers in evaluating CysLTRs markers on basophil membrane to improve cellular tests used for allergy diagnosis. During activation, basophils express a group of membrane molecules, which should provide clues about the role of these cells in the allergic inflammation, if selected as primary markers in a flow cytometry assay, such as the basophil activation test (BAT). While most common markers probing activated basophils are CD63 and CD203c, these molecules change their membrane expression directly linked to cell activation and independently from an IgE- or a non-IgE-mediated stimulus. Newly incoming markers might help researchers in differentiating the role of basophils in innate or acquired immunity. For example, the BM16 monoclonal antibody, which reacts with CD294, the prostaglandin D2 receptor, may give insights on the role of PGD_2_ in allergic inflammation, as PGD_2_ metabolites are well recognized markers of allergy, particularly for mast cells (16, 17). The search for new target molecules, either for allergy treatment or lab investigation, is a major goal for the development of novel approaches in diagnosis and therapy (18). In addition, the 2-oxoglutarate receptor 1 (OXGR1), a new cysteinyl receptor called receptor of the CysLT_E_ or GPR99, is expressed in mast cells and its ligand LTE_4_ is able to recruit both eosinophils and basophils to the inflammatory site in asthma (19). Products from 5-lipooxygenase regulate basophil migration (5-oxo-ETE) and degranulation (LTB_4_) (20), then the role of these mediators in chronic inflammation involving basophils should be reappraised. CysLTs, particularly CysLT1, activate the recruitment of both alpha–beta and gamma–delta effector T cells to the inflamed tissue (21). During allergy, T cells increase their expression of CyLTRs (22), an evidence that would suggest the fundamental role exerted by Cys-leukotrienes in the relationship basophils/T cells, during inflammation.

The conclusion, which the authors reached, describes a landscape where basophils play a strategic role at the cross-road of innate and acquired immunity, and in this sense, they reported a commonly accepted overview of the problem (23). Further insights about the function of lipid mediators in this complex basophil/T cell interplay might improve our knowledge and comprehension about the role of basophils in chronic inflammation.

## Author Contributions

The author confirms being the sole contributor of this work and approved it for publication.

## Conflict of Interest Statement

The author declares that the research was conducted in the absence of any commercial or financial relationships that could be construed as a potential conflict of interest.
